# Modelling the odor profile of fungal Solid-State Fermentation

**DOI:** 10.1038/s41538-025-00627-0

**Published:** 2026-06-19

**Authors:** Juan F. Sandoval, Jane K. Parker, Joe Gallagher, Julia Rodriguez-Garcia, Kerry Whiteside, David N. Bryant

**Affiliations:** 1 Institute of Biological, Environmental and Rural Sciences (IBERS), Aberystwyth University, Aberystwyth, SY23 3EE UK; 2 Department of Food and Nutritional Sciences, University of Reading, Whiteknights, Reading, UK; 3 Nutrition and Food Science Area, Preventive Medicine and Public Health, Food Science, Toxicology and Forensic Medicine Department, Faculty of Pharmacy, Universitat de València, Avda. Vicent Andrés Estellés, s/n, 46100 Burjassot, València, Spain; 4 Samworth Brothers Limited, Leicestershire, LE13 1GA UK

**Keywords:** Biochemistry, Biological techniques, Biotechnology, Microbiology, Plant sciences

## Abstract

The sensory properties of alternative proteins are key to consumer acceptance, yet the processes shaping their odor remain unclear. Solid-state fermentation (SSF), a promising method for producing alternative proteins from agro-industrial by-products such as bagasse, brans, pomaces, husks and oil cakes, is used in this study to model odor profile development of surplus bread crusts, supplemented with perennial ryegrass protein with *Rhizopus oligosporus*, *Aspergillus oryzae*, and *Neurospora intermedia* at 32 °C for up to 72 h. Volatile organic compounds (VOCs) were analyzed by solid-phase microextraction (SPME) followed by gas chromatography-mass spectrometry (GC–MS), identifying over 150 compounds. A mechanistic model based on the Weber–Fechner law predicted odor profiles from VOC concentrations, odor descriptors and thresholds, and was validated against a quantitative descriptive analysis (QDA) performed by a trained panel using multiple factor analysis (MFA). The model reflected changes in the overall odor intensity and sweet, baked, and grass-like notes, though correlations were weaker for fungal-derived descriptors (fruity, earthy, herbal). These findings elucidate how fungal SSF alters odor profiles in alternative proteins and establish a framework for mechanistic odor prediction in food systems.

## Introduction

The alternative proteins (AP) market is a fast-growing sector, with expected compound annual growth rates between 10% and 18.5% from 2021 to 2027 across high-income countries^1^. In this context, AP refers to protein sources considered alternatives to meat, and includes those derived from plants, insects, microbes, and cultured meat. Although global demand has driven rapid advancements across these categories, translating these innovations into commercially viable novel foods still proves challenging, leaving the AP sector as a relatively small fraction of the food market^2^.

Despite growing awareness of the environmental impact of traditional animal proteins, the cost and sensory attributes of AP still represent barriers for consumer acceptance^3^. Among these attributes, texture, odor, and flavor are the most crucial, requiring physical, chemical, and/or biological processing methods to align them with the cultural requirements of specific regions. For instance, the production of meat analogs from plant-based AP in Western countries requires intensive thermophysical processes, like extrusion, to mimic the fibrous texture, and the use of flavor-enhancing compounds to mimic its flavor profile^4^. Understanding the impact of these processes on sensory attributes is crucial for developing novel foods based on AP.

Solid-state fermentation (SSF), a fermentation method in which microorganisms are grown on solid substances in the absence or near absence of free water, has been shown to enhance the nutritional content and sensory attributes of diverse substrates. These include cereals, legumes, fruits, vegetables, as well as agro-industrial by-products such as brans, pomaces, and husks, and even food waste^5^. A key area of interest in SSF is the formation and modification of volatile organic compounds (VOCs), which are responsible for the complex odor profiles of foods. Several studies have reported changes in VOCs composition following SSF^6^, using a range of microorganisms, including yeasts (e.g., *Yarrowia* spp., *Saccharomyces* spp.), bacteria (e.g., *Bacillus* spp.), and filamentous fungi (e.g., *Trichoderma* spp., *Aspergillus* spp., *Neurospora* spp., *Rhizopus* spp.)^7,8^. The choice of microorganism and substrate is critical, as different species can result in markedly different VOCs composition even when grown on the same material^9^. For example, in a study comparing koji produced from the SSF of soybeans or wheat with either *Bacillus amyloliquefaciens* or *Aspergillus oryzae*, it was shown that *A. oryzae* favored the generation of aldehydes (2-methylpropanal, 2-methylbutanal, 3-methylbutanal), while *B. amyloliquefaciens* led to a more diverse set of carboxylic acids, alcohols, esters, ketones, furans, pyrazines, and phenols^8^.

The variety of VOCs that can be biosynthesized during SSF is diverse^10^. Techniques such as gas chromatography (GC), gas chromatography-olfactometry (GC-O), liquid chromatography (LC), and mass spectrometry (MS) have been used to identify the VOC composition of a given material and infer the presence of specific odor notes^11^. However, translating this information into a cohesive odor profile for an entire substrate remains a complex task, which is why trained sensory panels remain the gold standard for assessing the odor profile of foods. The AP industry requires innovation in these methods, as sensory panels are expensive and time consuming, and some of the microorganisms used in SSF are still being regulated in regions of the world, which limits their application in foods. For example, *Rhizopus oligosporus* is approved to commercially produce tempeh, a traditional Asian food, in the EU^12^, but *Neurospora intermedia*, though used in red oncom^13^, has not been approved.

Using analytical data to predict the odor profile of foods requires understanding the physicochemical interactions that occur between the VOC, perceptual effects in mixtures (e.g., suppression and configural perception), the surrounding media, and the human olfactory system. Due to the complexity of human perception, changes in the concentrations of VOCs are not linearly related to the intensity of the perceived odor. A model describing the relationship between the odor intensity (${\rm{OI}}$) and the intensity of the physical stimuli (VOCs concentration) (${\rm{I}}$) was proposed by Fechner in 1860, based on Weber’s law, known as Weber-Fechner’s law (Eq. 1)^14^:

1 $${\rm{OI}}={\rm{a}}\log \left({\rm{I}}\right)+{\rm{k}}$$

where ${\rm{k}}$ is a proportionality constant and ${\rm{a}}$ is the slope. The parameter ${\rm{a}}$ varies significantly based on the VOC, with a higher value indicating increased supra-threshold sensitivity to the stimulus. The intensity of the stimuli ${\rm{I}}$ can be related to the VOC concentration ${\rm{C}}$ and its odor threshold concentration C_0_^15^ so that the Weber-Fechner’s law is transformed to Eq. 2:

2 $${\rm{OI}}={\rm{a}}\log \left(\frac{{\rm{C}}}{{{\rm{C}}}_{0}}\right)+{\rm{k}}$$

The proportionality constant ${\rm{k}}$ is 0.5 by definition, from the understanding that if a VOC concentration ${\rm{C}}$ is at its threshold ${{\rm{C}}}_{0}$, $\sim$50% of sensory panelists will detect a smell, which would imply $\sim$50% would give a minimal intensity score of 1 and $\sim$50% a score of 0^16^. ${{\rm{C}}}_{0}$ values are not available for all VOC, even less so in the same medium, but are compiled in databases such as the one provided by Leffingwell & Associates^17^. The parameter ${\rm{a}}$, also known as the Weber-Fechner coefficient, is substance specific and has been measured for a relatively small number of VOCs of interest for air quality studies, such as the one carried out by the German committee on indoor air guide values, reporting ${\rm{a}}$ as low as 1.95 for acetic acid and as high as 3.31 for 2-methylnaphthalene^18^. This equation has received widespread use when studying air and water quality, as the ${{\rm{C}}}_{0}$ and ${\rm{a}}$ data for the most analyzed VOCs is available, and the final objective is an overall OI. Due to the nature of the model, it fails to predict OI for VOCs at concentrations below the ${C}_{0}$, which is where other models such as Steven’s power law have been proposed^19^ and found to provide better estimations of the magnitude of the intensity^20^, but being more sensitive to VOC specific parameters and thus requiring their calculation to be used properly.

Application of these concepts has been reported in dairy alternatives^21^ and mineral oils^22^ to predict simplified odor profiles and chemical parameters of the VOCs. Prediction of overall odor profiles has been attempted through numerical correlation methods, such as machine learning models combining analytical and sensory panel data^23^, which results in mathematical models that are mostly substrate-specific. A mechanistic approach that combines these data and phenomenological understanding of odor generation is needed to create more generalized models.

Thus, a novel mechanistic model based on Weber-Fechner’s law is proposed, incorporating analytical data (semi-quantitative mass concentration of compounds via solid-phase micro-extraction (SPME) GC-MS) and the odor descriptors of individual VOCs to predict the odor profile of fermented foods. The model was applied to the resulting SSF of surplus bread crusts and perennial ryegrass substrates with filamentous fungi *Aspergillus oryzae, Neurospora intermedia*, and *Rhizopus oligosporus*, a process which has previously been reported to improve the nutritional quality of the combined substrate^24^ and to change its VOC composition^25^. The resulting odor profiles were compared to a quantitative descriptive analysis (QDA) by trained sensory panelists of the same SSF materials, demonstrating the model’s capacity to predict specific descriptors and highlighting the requirements for improvement in future work.

## Results and discussion

### Identification of volatile organic compounds

A total of 156 VOCs were identified across all SSF experiments (Table 1). The compounds were classified according to their functional chemical group resulting in a total of 17 alcohols, 25 aldehydes, 21 esters, 11 furans, 14 ketones, 4 organic acids, 4 phenols, 9 pyrazines, 1 pyridine, 6 pyrroles, 5 sulfur compounds, 28 terpenes, 7 dioxolanes and 4 compounds that did not fit the other groups. When classified by their likely source of origin, there were 29 products typical of lipid oxidation, 41 products associated with Maillard reactions, 32 likely to be derived from PRG, 49 likely to have been produced during SSF, and 5 that did not fit into any of the other groups (Table A1).

**Table 1:** Volatile compounds in solid-state fermented (SSF) surplus bread crusts and perennial ryegrass with Aspergillus oryzae, Neurospora intermedia and Rhizopus oligosporus

n.	Compound name	LRIᵃᵇ	Odor categoriesᶜ	Odor threshold in water (μg/kg)ᵈ	IDᵉ
**Alcohols**					
1	2-methylpropanol	623	alcohol, baked, fermented	7.00E + 03	A
2	butanol	659	alcohol	3.80E + 02	A
3	1-penten-3-ol	680	sulfurous	4.00E + 02	A
4	3-methylbutanol	733	alcohol, fruit	2.75E + 02	A
5	2-methylbutanol	736	sweet	3.20E + 02	A
6	pentanol	765	alcohol, green	4.00E + 03	A
7	2,3-butanediol 1	789	dairy	1.00E + 05	A
8	2,3-butanediol 2	793	dairy	1.00E + 05	A
9	(Z)-3-hexen-1-ol	851	green	4.48E + 02	A
10	2-hexen-1-ol	866	green	4.29E + 04	A
11	hexanol	868	green, rancid	2.50E + 03	A
12	heptanol	969	alcohol, green	3.00E + 00	A
13	2-octanol	999	floral	9.58E-02	A
14	benzyl alcohol	1038	floral	1.00E + 04	A
15	octanol	1070	rancid	1.10E + 02	A
16	phenylethyl alcohol	1120	floral	1.10E + 03	A
17	(E)-3-nonen-1-ol	1171	green, vegetable	5.06E + 03	B
**Aldehydes**					
1	methylpropanal	562	baked, sweet	1.50E + 00	A
2	2-butenal	648	sweet		A
3	3-methylbutanal	651	baked, sweet	1.10E + 00	A
4	2-methylbutanal	661	baked, sweet	1.00E + 00	A
5	pentanal	698	fermented, sweet	1.20E + 01	A
6	2-methyl-2-butenal	743	nut, sulfurous	4.35E + 02	A
7	(E)-2-pentenal	753	fruit, rancid	1.50E + 03	A
8	3-methyl-2-butenal	785	nut	4.74E + 02	A
9	hexanal	800	green	4.50E + 00	A
10	(Z)-3-hexenal	800	green	4.50E + 00	A
11	(E)-2-hexenal	854	green	1.70E + 01	A
12	(Z)-4-heptenal	900	rancid	8.00E-01	A
13	(E)-2-heptenal	958	rancid	1.30E + 01	A
14	phenylacetaldehyde	1050	floral	4.00E + 00	A
15	(E)-2-octenal	1061	fruit, rancid	3.00E + 00	A
16	5-methyl-2-isopropyl-2-hexenal	1092	green, sweet		B
17	nonanal	1106	fruit, rancid	1.00E + 00	A
18	(E)-5-methyl-2-isopropyl-2-hexenal	1112	floral, green		A
19	(E,E)-2,6-nonadienal	1157	rancid, vegetable	1.14E-02	A
20	(E,Z)-2,6-nonadienal	1163	rancid, vegetable	1.14E-02	A
21	(E)-2-nonenal	1167	rancid	8.00E-02	A
22	(E)-2-decenal	1266	herb	4.00E-01	A
23	α-ethylidenbenzeneacetaldehyde	1283	baked, fermented, nut, sweet, vegetable		B
24	(E)-2-undecenal	1368	herb	4.10E-02	A
25	5-methyl-2-phenyl-2-hexenal	1503	sweet		A
**Esters**					
1	ethyl acetate	613	alcohol	8.29E + 02	A
2	ethyl lactate	798	fruit	1.40E + 04	A
3	propyl propanoate	806	fruit	5.70E + 01	A
4	ethyl 2-butenoate	844	fruit		A
5	ethyl hexanoate	998	fruit	1.00E + 00	A
6	2-hexenyl acetate	1018	fruit	1.55E + 00	A
7	ethyl (E)-2-hexenoate	1044	spice, sweet		A
8	2-methylbutyl butanoate	1059	fruit	2.78E + 00	A
9	ethyl heptanoate	1097	fruit	2.20E + 00	B
10	ethyl 4-octenoate	1190	fruit		B
11	ethyl octanoate	1196	fruit	1.47E + 02	A
12	ethyl 3-octenoate	1198	fruit		B
13	ethyl (E)-2-octenoate	1246	fruit		B
14	β-phenethyl acetate	1263	dairy	2.16E + 03	A
15	α-terpinyl acetate	1353	herb		A
16	neryl acetate	1359	floral	9.00E + 00	A
17	2-phenylethyl propanoate	1360	sweet		B
18	ethyl (Z)-4-decenoate	1380	fruit		B
19	ethyl decanoate	1394	fruit, rancid	5.10E + 02	A
20	citronellyl butyrate	1534	floral, fruit		A
21	geranyl butyrate	1562	floral	6.28E-01	A
**Furans**					
1	2-methylfuran	606	alcohol, sweet	3.50E + 03	A
2	3-methylfuran	638	fermented		A
3	2-ethylfuran	703	baked	6.77E-01	A
4	2-vinylfuran	725	coffee	3.38E + 01	A
5	2-furfural	835	nut	2.77E + 02	A
6	2-furanmethanol	860	alcohol, sweet	2.04E + 01	A
7	5-methyl-2(3H)-furanone	870	fermented, green		A
8	2-methyl-5-propylfuran	888	vegetable		B
9	2-acetylfuran	914	coffee, sweet	2.23E + 04	A
10	5-methyl-2(5H)-furanone	940	spice, sweet		B
11	2-pentylfuran	994	baked, earthy	4.46E-02	A
**Ketones**					
1	butanedione	589		3.00E + 02	A
2	2-butanone	599	alcohol	5.00E + 04	A
3	3-methyl-2-butanone	656	misc.	4.41E + 02	A
4	1-pentene-3-one	686	green, seaweed	1.00E + 00	A
5	2,3-pentanedione	695	dairy	3.00E + 01	A
6	3-hydroxybutanone	710	dairy	8.00E + 02	A
7	4-cyclopentene-1,3-dione	887	sweet		A
8	2-heptanone	891	fruit	1.40E + 02	A
9	2-methyl-2-cyclopentenone	907	spice, sweet		A
10	3-methyl-4-heptanone	929	fruit, nut	5.36E + 02	B
11	2-methyl-6-heptanone	956	fruit		A
12	2,3-octanedione	984	green, rancid		A
13	6-methyl-5-heptene-2-one	988	fruit	5.00E + 01	A
14	2-hydroxy-3-methyl-2-cyclopenten-1-one	1029	baked	9.52E + 02	A
**Organic acids**					
1	acetic acid	597	vinegar	1.61E + 05	A
2	2-methylpropanoic acid	750	dairy	4.03E + 03	A
3	3-methylbutanoic acid	833	dairy	1.30E + 02	A
4	2-methylbutanoic acid	846	dairy	5.00E + 01	A
**Phenols**					
1	4-vinylphenol	1219	misc.	1.00E + 01	A
2	p-vinylguaiacol	1324	spice	2.63E + 04	A
3	eugenol	1368	spice	6.00E + 00	A
4	methylisoeugenol	1501	spice	1.86E + 04	A
**Pyrazines**					
1	methylpyrazine	826	nut	6.00E + 04	A
2	2,5-dimethylpyrazine	916	nut	8.00E + 02	A
3	ethylpyrazine	918	baked, sweet	6.00E + 03	A
4	2,3-dimethylpyrazine	922	baked, nut	2.50E + 03	A
5	2-isopropylpyrazine	972	baked		A
6	2-ethyl-5-methylpyrazine	1001	nut, sweet	1.00E + 02	A
7	2,6-diethylpyrazine	1083	baked, nut	4.31E + 03	A
8	2-ethyl-3,6-dimethylpyrazine	1083	baked, earthy	2.59E + 01	A
9	2-methyl-3,5-diethylpyrazine	1163	baked, nut		B
**Pyridines**					
1	2-acetylpyridine	1038	baked	1.90E + 01	A
**Pyrroles**					
1	1-ethylpyrrole	816	cooked		A
2	2-formylpyrrole	1008	cooked	1.25E + 07	A
3	1-ethyl-2-formylpyrrole	1057	cooked		B
4	2-acetylpyrrole	1062	baked	1.70E + 05	A
5	1-(3-methylbutyl)pyrrole	1063			A
6	1-furfurylpyrrole	1189	earthy, vegetable	9.35E + 02	A
**Terpenes**					
1	α-phellandrene	1011	herb, misc.	5.00E + 02	A
2	p-cymene	1031	herb	1.14E + 01	A
3	d-limonene	1036	citrus	1.00E + 01	A
4	2,2,6-trimethylcyclohexanone	1042	floral	1.16E + 02	B
5	terpinolene	1095	earthy	2.00E + 02	A
6	linalool	1101	citrus, floral	6.00E + 00	A
7	α-cyclocitral	1128	fruit, floral	2.51E + 00	A
8	α-terpineol	1200	citrus, herb	3.30E + 02	A
9	safranal	1211	herb		A
10	β-cyclocitral	1233	fruit, misc.	5.00E + 00	A
11	β-cyclohomocitral	1271	spice		A
12	dihydroedulan	1306	earthy		B
13	α-copaene	1390	earthy	6.23E-03	A
14	α-santalene	1426	earthy		A
15	caryophyllene	1444	earthy, misc.	6.40E + 01	A
16	6,10-dimethyl-5,9-undecadien-2-one	1458	floral	1.27E + 01	A
17	(E)-β-farnesene	1462	fruit		A
18	β-chamigrene	1475	earthy		B
19	alpha-humulene	1478	baked	1.20E + 02	A
20	β-curcumene	1492	spice		B
21	α-cuparene	1495			B
22	dehydro-β-ionone	1497	earthy	7.00E-03	B
23	β-bisabolene	1507	earthy		A
24	(Z)-α-bisabolene	1513	earthy		A
25	cubebol	1521	spice		A
26	dihydroactinolide	1535	earthy, spice	6.57E + 03	B
27	(Z)-nerolidol	1538	citrus, floral	1.00E + 01	A
28	santalol	1697	earthy	2.43E + 02	A
**Sulfur compounds**					
1	dimethyl sulfide	534	seaweed	3.00E-01	A
2	dimethyl disulfide	747	vegetable	6.08E + 00	A
3	methional	908	baked	2.15E + 01	A
4	5-methyl-2-formylthiophene	1131	baked, nut	6.99E + 01	A
5	3-methyl-2-formylthiophene	1135	cooked		A
**Acetals of 2,3-Butanediol**					
1	4,5-dimethyl-2-isopropyl-1,3-dioxolane 1	876			A
2	4,5-dimethyl-2-isopropyl-1,3-dioxolane 2	912			A
3	4,5-dimethyl-2-isopropyl-1,3-dioxolane 3	920			A
4	2-isobutyl-4-methyl-1,3-dioxolane	952	fruit		A
5	4,5-dimethyl-2-isobutyl-1,3-dioxolane 1	975			A
6	4,5-dimethyl-2-isobutyl-1,3-dioxolane 2	1005			A
7	4,5-dimethyl-2-isobutyl-1,3-dioxolane 3	1015			A
**Others**					
1	p-xylene	880	misc.		A
2	nonane	899	misc.	1.55E + 02	A
3	benzaldehyde	966	dairy	3.50E + 02	A
4	1,1,6-trimethyl-1,2-dihydronaphthalene	1372	misc.		A

ᵃ Linear retention index on a DB-5 column, calculated from a linear equation between each pair of straight chain alkanes C6–C20.

ᵇ Comparison LRIs and their source can be found in the supplementary data (Table A1).

ᶜ Odor categories reduced from odor descriptors. Complete information can be found in the supplementary data (Table A1).

ᵈ Sources for the odor thresholds can be found in the supplementary data (Table A1).

ᵉ A: Mass spectra and authentic LRI match, B: Mass spectra and external LRI match.

Analysis of the semi-quantitative concentration of these compounds in SSF samples revealed the origins of key VOCs that were discussed in prior work^25^ (Table A3). For example, several VOCs known to occur in BC, such as 2-methylfuran and 2,5-dimethylpyrazine, and in PRG, such as phenylacetaldehyde and dimethyl sulfide, were identified. All fungi generated markers of fungal growth, including 2,3-butanediol, 2-methylpropanol, 2-methylbutanol, 3-methylbutanol and 2-butenal^26^, as well as compounds typically associated with Maillard reactions, such as 3-methylbutanal, 2-methylbutanal, benzeneacetaldehyde and 2-methylpropanal. Fungi-specific metabolites were also observed, including 2-methylbutanoic acid and 2-methylpropanoic acid by AO, and a diverse set of esters, terpenes and sesquiterpenes by NI and RO. Conversely, several VOCs related to PRG, such as hexanal, dimethyl sulfide, dimethyl disulfide, (Z)-3-hexenal and 1-penten-3-one, decreased in abundance during SSF. Although these results suggest that the odor profile of the substrates would change over SSF time, the principles modeled by Weber-Fechner’s law^27^ warrant further analysis of the data to understand how and in what magnitude.

### Properties of identified volatile organic compounds

The construction of the model required an initial compilation of properties of the VOCs identified. The dimensionless Henry’s volatility constant ${{\rm{H}}}_{{\rm{V}}}^{{\rm{CC}}}$, required to adjust the air-phase measurements to semi-quantitative concentrations in the water solutions (Eq. 3), was readily available for 63 out of 156 VOCs identified from Sander^28^. The rest were calculated with the EPI suite software with the group and bond contribution methods, estimation models with high accuracy (${r}^{2}$ > 0.92) based on the chemical structure of the molecules^29^ (Table A1).

Odor thresholds were found reported for 108 of the identified VOCs, in either air or water. A median was calculated for cases in which multiple reports and/or replicates of the odor thresholds existed. The median statistic was chosen instead of a mean average due to the high variability in the order of magnitude encountered between reports of the same molecules, as evidenced by the odor threshold of benzaldehyde, reported as 3 ppb^30^, 350 ppb^31^, and 3500 ppb^32^ in water solutions. The variability arises from the measurement method used for these thresholds, which depends on the sensory panelists. It is a well-documented issue in the field^33,34^, caused by various factors such as gender, age, race, and body type, and known to fluctuate between different testing days^35^.

Alternatively, several predictive models for odor thresholds have been developed, utilizing diverse molecular descriptors. These include models based on physicochemical properties such as polarity, hydrogen bonding capacity, and gas–liquid partitioning (*R*^2^ = 0.76)^36^, quantitative structure–activity relationship (QSAR) approaches using Monte Carlo simulations (*R*^2^ > 0.61)^37^, and models incorporating mass transfer-related properties such as saturation pressure and partition coefficients (*R*^2^ = 0.77)^38^. However, all require data that is not readily available for the same VOCs, which did not have a reported threshold.

The 48 compounds without reported odor thresholds were excluded from the study, as it is not possible to calculate their odor activity values or include them in the mechanistic model without threshold data, making their contribution to odor profiles unverifiable. These included 9 esters produced by NI and RO in both BC + G and BC + W substrates, generally associated with fruity, floral, and ethereal odors, and 11 terpenes and sesquiterpenes, initially considered exclusive to PRG, but found to be synthesized by NI and RO, typically linked to earthy and spice odors. Also excluded were 12 compounds associated with Maillard reactions and 6 related to lipid degradation.

The VOCs included in the analysis had 64 unique odor descriptors related to them. While the mechanistic model was fundamentally capable of predicting an odor intensity (OI) for each of these descriptors, the individual contribution of most of them was insignificant due to the concentration of the VOCs that made of said descriptors being smaller than their odor thresholds ($C < {C}_{0}$). The odor descriptors were thus grouped into categories based on their correlation (Table A2), the insights of expert flavorists and the trained sensory panel, resulting in 21 distinct categories: alcohol, baked, citrus, coffee, cooked, dairy, earthy, fermented, floral, fruit, green, herb, miscellaneous, nut, rancid, seaweed, spice, sulfurous, sweet, vegetable, and vinegar (Table A4). The categories coffee, green, floral, fruit, seaweed, and vinegar were mostly made up of descriptors of the same name, as they were either frequent amongst the VOCs or did not fit in the other categories. Categories such as alcohol (ethereal, fusel, rum and alcoholic), dairy (butter, cheese, coconut), earthy (woody, earthy, musk, mushroom, pine and orris) and herb (herbal, cilantro and terpenic), grouped thematically similar descriptors. The category miscellaneous (medicinal and burnt plastic) grouped descriptors that did not fit the other categories.

The most frequent odor categories were fruit (23 VOCs), sweet (18 VOCs), baked (16 VOCs), green (14 VOCs) and rancid (14 VOCs), while the least frequent were seaweed (2 VOCs), sulfurous (2 VOCs), coffee (2 VOCs) and vinegar (1 VOCs). The frequency of categories across the SSF samples was not indicative of their OI, as this was based on the VOCs with the lowest odor thresholds and relatively high abundance.

### Mechanistic modeling of odor profiles

**Fig. 1:**
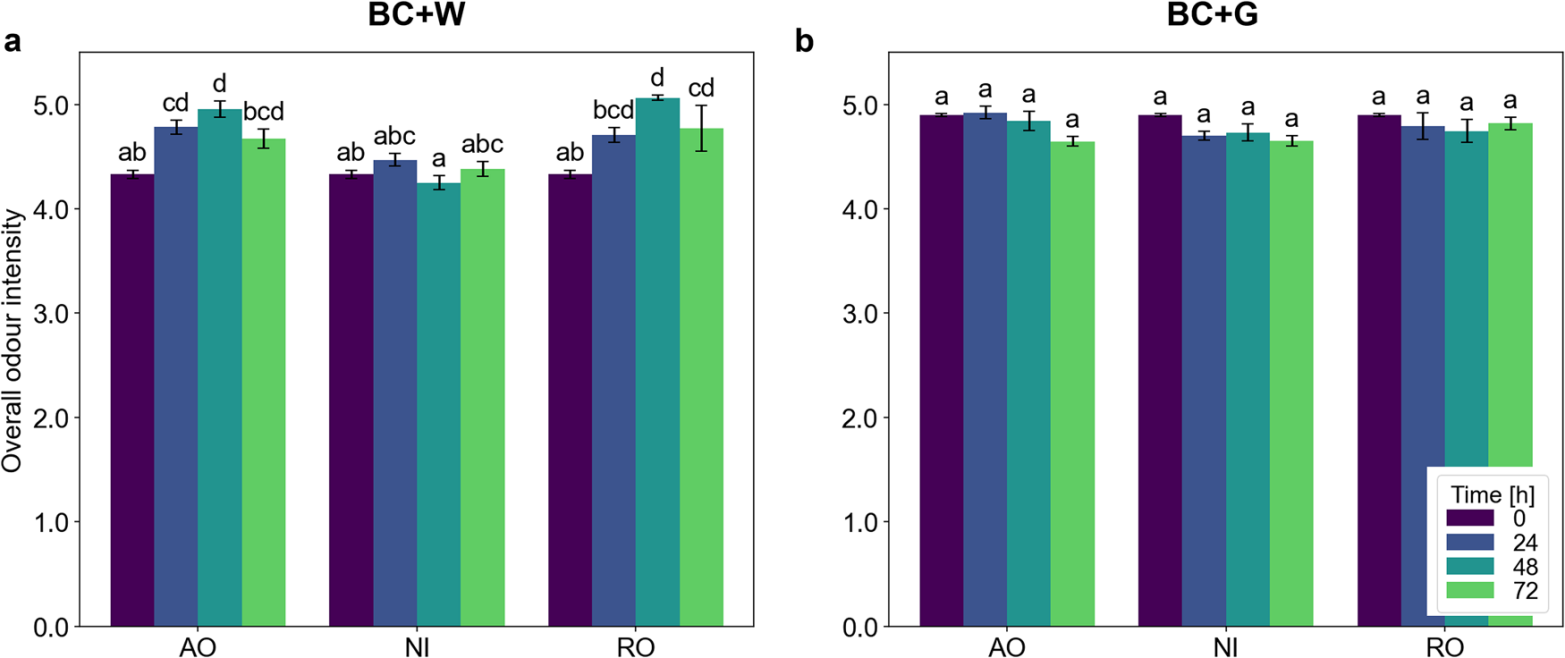
Predicted overall odor intensity. Predicted overall odor intensity based on the concentration of volatile organic compounds at 0, 24, 48 and 72 h of solid-state fermentation (SSF) for Aspergillus oryzae (AO), Neurospora intermedia (NI) and Rhizopus oligosporus (RO) in **a** bread crusts + water (BC + W) and **b** bread crusts + perennial ryegrass (BC + G) substrates. Bars represent the mean value (*n* = 3) and vertical lines the standard error. Data with the same letters in the substrate group are not significantly different (*p* = 0.05), as determined by Tukey´s HSD post-hoc test.

Figure 1 shows the overall OI based on the concentration and odor thresholds of VOCs, based on the sum of the OAV of each VOC in each sample with the Weber-Fechner model (Eq. 6), at 0, 24, 48, and 72 h of SSF with AO, NI, and RO in both BC + W and BC + G substrates. In B&W substrates (Fig. 1a), SSF significantly increased the OI over time (*p*-value < 0.001), with RO and AO achieving the highest overall OI after 48 h. In BC + G substrates (Fig. 1b) Neither fungi nor SSF time had a significant effect on the overall OI (*p*-value = 0.3). Although 108 VOCs were analyzed, 80 had average OAVs lower than one across all samples, with orders of magnitude ranging from 10^-1^ to 10^-14^; thus, their impact on the overall OI was of 0 due to the logarithmic scale. In contrast, the 28 VOCs with OAVs higher than 1, with orders of magnitude ranging from 10^1^ to 10^5^, exerted a predominant influence on the calculations. This implies that these VOCs dominate the volatile stimulus space of the SSF samples and are responsible not only for its overall OI, but also for the impact in each odor category. Similarly, VOCs with unique odor descriptors and low OAVs only had a minimal effect on the model predictions, resulting in a reduced list of relevant odor categories.

**Fig. 2:**
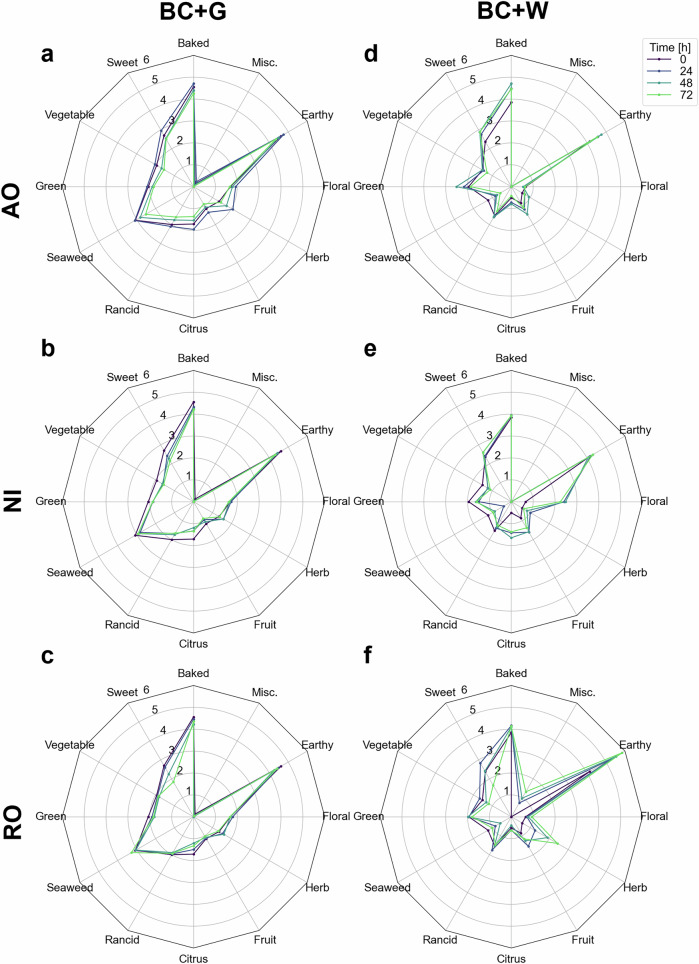
Predicted odor profiles. Predicted odor profiles based on the concentration of volatile organic compounds at 0, 24, 48, and 72 h of solid-state fermentation (SSF) for *Aspergillus oryzae* (AO), *Neurospora intermedia* (NI) and *Rhizopus oligosporus* (RO) in bread crusts + water (BC + W) and bread crusts + perennial ryegrass (BC + G) substrates. **a** AO in BC + G. **b** NI in BC + G. **c** RO in BC + G. **d** AO in BC + W. **e** NI in BC + W. **f** RO in BC + W. Each data point represents the mean value (*n* = 3) of the odor intensity in the category on a logarithmic scale.

Figure 2 shows the OI of the predicted odor categories (Eq. 8). Of the initial list, categories alcohol, coffee, cooked, dairy, fermented, nut, spice, sulfurous, and vinegar were removed from the study as their OI was close to 0 across all samples. These categories consisted of VOCs whose OAVs were too low to have an impact. For instance, the category coffee was represented by VOCs 2-vinylfuran and 2-acetylfuran and vinegar by acetic acid, all of which were significantly less abundant than their respective odor thresholds. The remaining categories, baked, earthy, floral, herb, fruit, citrus, rancid, seaweed, green, vegetable, sweet, and miscellaneous, had OI between 0 and 6. It is important to note that although common fermentation-related categories such as alcohol and fermented did not contribute meaningfully to the model, the quantitative descriptive analysis (QDA) performed by the trained panel did report a fermented category. This is consistent with the fact that fermented is a broad sensory descriptor, which in practice is made up of a complex combination of other categories (e.g., rancid, sweet, and earthy,). Moreover, the character of fermented differs substantially across substrates and microorganisms, meaning it is not always directly linked to the specific VOCs with the highest OAV in the model.

Across all SSF samples, the categories with the highest OI were baked (ranging from 3.74 to 4.71) and earthy (ranging from 3.94−5.84). Although 16 VOCs shared odor descriptors related to the baked category (bread, biscuit, hops, toasty, etc.), the contribution of 2-ethylfuran, 2-pentylfuran, methylpropanal, 2-methylbutanal, and 3-methylbutanal made up most of the category in all samples. These compounds have been previously reported to be abundant in bread, originating from Maillard reactions^7^, and were identified in the unfermented samples^25^. In BC + W SSF (Fig. 2d–f) their abundance increased over time, as noted from the capacity of the fungi to synthesize these compounds^25^, which translates into an increased OI in the baked category. AO SSF has the highest increase in the category over SSF time due to the relatively higher biosynthesis of 2-pentylfuran, 2-methylbutanal, and 3-methylbutanal, volatiles known to be produced by AO^8^. In BC + G SSF (Fig. 2a–c) the opposite occurs, and the category is reduced, suggesting different metabolic pathways in the presence of PRG. Moore and Lloyd^39^ similarly reported that in *Aspergillus* spp., the VOC profile varied markedly between growth on synthetic media and on corn varieties, with 2-pentylfuran notably absent in the synthetic media. The earthy category was primarily composed of 2-pentylfuran and α-copaene in all samples, and dehydro-β-ionone in the BC + G samples.

There were notable differences between BC + G and BC + W SSF, with only the categories fruit and green not being statistically different (*p*-value > 0.05). This is attributable to VOCs originating from PRG, which included odor descriptors related to the categories of vegetable, green, seaweed, and rancid. These categories are generally perceived as unpleasant in the context of plant-based foods and AP products. For example, vegetable and green notes have been shown to reduce consumer acceptance of plant-based foods, motivating extensive research into physical, chemical, and biological strategies to mitigate them^40^. The seaweed odor, related here to marine and fishy descriptors, has previously been linked to sulfurous descriptors in alliaceous foods and can be desirable in seafood and seafood alternatives^41^, but not in others. Rancid odors, typically arising from lipid degradation, are among the most widespread off-flavors in food^42^. The most impactful VOCs were hexanal (green), (Z)-3-hexenal (green), (E, E)-2,6-nonadienal (vegetable and rancid), (E, Z)-2,6-nonadienal (vegetable and rancid), dimethyl sulfide (seaweed) and dimethyl disulfide (vegetable), all known VOCs of *Lolium sp*^43^. These VOCs were present in all samples, but had higher OAVs in BC + G samples, which decreased over the SSF time.

Additionally, fungi-specific VOCs with OAVs >1 also led to unique changes in odor categories. In the BC + W samples, this had a significant effect (*p*-value < 0.05) in all categories except for fruit and rancid, while in BC + G samples, a significant effect (*p*-value < 0.05) in all categories except for fruit, green, herb and floral. This included 2-methylbutanoic acid and 2-hexenyl acetate in AO, 2-octanol, geranyl butyrate and ethyl hexanoate in NI, and α-copaene and caryophyllene in RO. In AO SSF, the sharp increase in 2-methylbutanal and 3-methylbutanal significantly increased the OI of the baked and sweet categories in BC + W. AO also increased the abundance of (E)-2-decenal in BC + G, which increased the OI of the herb category. NI SSF in BC + W had a higher floral OI from the rise in 2-octanol and geranyl butyrate. The impact on this category in NI, and others related to its unique VOCs such as fruit, is likely underestimated, as several other esters were uniquely biosynthesized in NI SSF, but their odor thresholds were not available. RO SSF in BC + W had a higher earthy OI, from the increase of α-copaene and caryophyllene, and a higher herb OI, from increased (E)-2-undecenal. These VOCs led to unique odor profiles for each fungi (Fig. 2), with AO enhancing the characteristic baked and sweet profile of bread, NI developing an ester-rich enhanced floral, fruit, and citrus profile, and RO a terpene-rich enhanced earthy and herb profile.

The pattern observed, where a few volatiles dominate the overall odor profile despite the presence of many others, reflects a common phenomenon observed in most food systems. Dunkel, et al.^44^ extensively characterized this effect, identifying 226 key food odorants (KFOs), from over 10000 VOCs, in 227 food and beverage samples. Of the 28 VOCs with OAVs >1 in this study, 17 were also in the list of KFOs: dimethyl sulfide, dimethyl disulfide, methylpropanal, 2-methylbutanal, 3-methylbutanal, hexanal, (Z)-3-hexenal, (Z)-4-heptenal, ethyl hexanoate, D-limonene, nonanal, (E)-2-nonenal, (E)-2-decenal, 2-hexenyl acetate, (E,E)-2,6-nonadienal, (E,Z)-2,6-nonadienal and (E)-2-undecenal. Most of these compounds are aldehydes related to Maillard reactions and lipid degradation, making them common in bakery products and cereals^7^, and have relatively low odor thresholds, ranging from 0.01−10 µg/kg (with a mean value of 8.7 µg/kg), relative to the overall range of 0.01 to 1.25 × 107 µg/kg of all VOCs in the study (with a mean value of 1.25 × 105 µg/kg). The remaining 11 VOCs with OAVs >1 were 2-ethylfuran, 2-pentylfuran, 2-octanol, p-cymene, 2-methylbutyl butanoate, α-copaene, caryophyllene, α-humulene, dehydro-β-ionone, (Z)-nerolidol, and geranyl butyrate. Most of these compounds were related to PRG or synthetized by AO, NI or RO in SSF and thus are not common KFOs identified in other foodstuffs, aside from the furans, which are found in cereals^45^. Of the 48 compounds that were previously excluded from the analysis due to the lack of odor threshold data, none are found within the list of KFOs, suggesting that most of them would not be odor active as their threshold might otherwise have been reported.

Overall, the model estimates differences in the odor profiles of the samples, which vary with the substrate, the fungus, and the SSF time. Comparing the unfermented substrates (Fig. 2, time 0), it can be observed that the addition of PRG to BC increases the unpleasant vegetable, green, seaweed, and rancid categories, but also the pleasant floral, herb, fruit, and citrus categories, while reducing the characteristic baked category of bread. SSF in the presence of PRG has been shown to slow down fungal growth^24^, thereby reducing the magnitude of the impact of SSF on the odor profile of the BC + G substrates. Nonetheless, AO and NI reduced the negative odor categories over time, suggesting that fungal SSF can reduce the intensity of unpleasant odors in forage crops. In contrast, SSF in BC + W substrates leads to unique changes in the odor profiles with each fungus, showing the impact of their diverse metabolisms over odor modulation.

Rather than serving as a tool for precise quantitative prediction, the current model is best understood as a means of discerning patterns, beyond simply cataloging the presence or absence of VOCs. A key limitation of the model lies in its treatment of VOC mixtures, as olfactory perception is not a linear summation of individual compound intensities, but rather the result of intricate interactions^46^. Additionally, human olfaction is highly contextual and influenced by cognitive and environmental factors^47^. Despite these constraints, the model logically highlights potential KFOs that could be the focus of future research. To evaluate the model’s reliability, a QDA was conducted using a trained sensory panel. This enabled a direct comparison between the predicted and perceived odor profiles of the SSF samples.

### Quantitative descriptive analysis (QDA)

Across six training sessions designed to develop a consensus vocabulary, the panel identified a total of 51 unique odor descriptors. These individual descriptors, each reported by between 1 and all 13 panelists, were consolidated into 16 broader odor categories to ensure consistency in analysis (e.g. bread and bread crusts descriptors combined into the cereal category), and the panelists were asked to identify these categories. Several of these categories were not identified by a majority of the group (>7 of the 13 panelists) and were taken out of the study, those were dairy (milk), earthy, woody (wood and hardwood), brown fruit, herb (mixed herbs and oregano), spice (curry, turmeric, pepper and liquorice), sulfurous and vegetable. The final odor categories (Table 2) were cereal (bread, bread crusts, malt, grain, cereal and barley), fermented (soy sauce, musty and silage), rancid (linseed oil), seaweed (nori seaweed), grass (dried grass, hay, green tea, meadow and chamomile), sweet grass (fresh grass), molasses (molasses, treacle, caramel and maple syrup) and malt extract (malt extract, barley extract, marmite and yeast extract). It is important to note that these panel-derived categories were treated as a separate dataset from the mechanistic model to avoid overfitting the data and to provide a transparent comparison that highlights where the model converges and diverges from human perception.

**Table 2:** References for the odor categories in solid-state fermented (SSF) surplus bread crusts and perennial ryegrass with Aspergillus oryzae, Neurospora intermedia and Rhizopus oligosporus

Odor category	Reference	Brand and supplier
Cereal	Bread crusts	Bradgate bakery (Samworth Brothers Ltd, Leicestershire, UK)
Fermented	Soy sauce	Kikkoman (Kikkoman Foods Europe BV, Düsseldorf, Germany)
Rancid	Linseed oil	Natco (Natco Foods Ltd, Buckingham, UK)
Seaweed	Nori seaweed	Yutaka (Tazaki Foods Ltd, Enfield, UK)
Grass	Fresh grass, dried at 50 C for 48 h	Local supplier
Sweet grass	Fresh grass	Local supplier
Molasses	Molasses	Meridian (Meridian Foods Ltd, Belfast, UK)
Malt extract	Malt extract	Meridian (Meridian Foods Ltd, Belfast, UK)

Due to the lack of consensus among panelists regarding the earthy odor category during the training sessions, the attribute was further investigated. To explore this, a supplementary blind smell trial was conducted using five chemical standards commonly associated with earthy and moldy odors: 2-nonenal, geosmin, 2,4,6-trichloroanisole, 2,6-dichlorophenol, 2-isopropyl-3-methoxypyrazine, and 2-methylisoborneol. Interestingly, panelists rarely used earthy descriptors to describe these standards; instead, they identified them with terms such as “moldy,” “chemical,” and “leathery.” These findings suggest that descriptors related to the fermented odor category may have been used more frequently by panelists to capture earth-like qualities, which are known to be adjacent sensory perceptions^48^, particularly in BC + G samples.

**Fig. 3:**
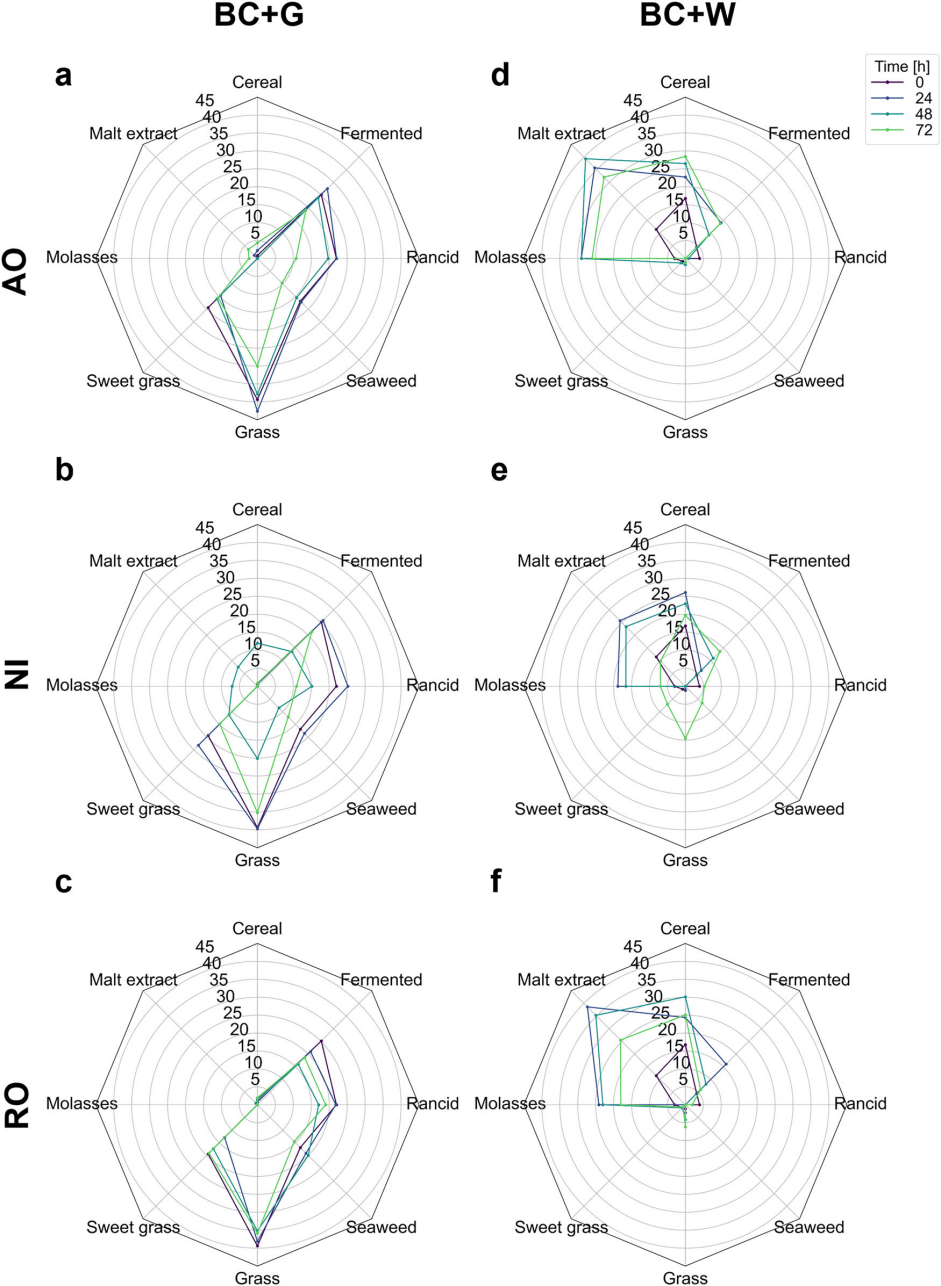
Sensory panel odor profiles. Odor profiles from trained sensory panels at 0, 24, 48, and 72 h of solid-state fermentation (SSF) for Aspergillus oryzae (AO), Neurospora intermedia (NI), and Rhizopus oligosporus (RO) in bread crusts + water (BC + W) and bread crusts + perennial ryegrass (BC + G) substrates **a** AO in BC + G. **b** NI in BC + G. **c** RO in BC + G. **d** AO in BC + W. **e** NI in BC + W. **f** RO in BC + W. Scores in each category represent the mean value (*n* = 13).

Figure 3 shows the resulting scores of the QDA. MANOVA was performed after splitting the data by substrate, as the profiles were significantly different and overshadowed the statistical effect of the other variables SSF time and fungi (Table A5). This was mainly driven by the concentration of all VOCs originating from PRG (Table A3), mainly terpenes such as β-cyclocitral and β-cyclohomocitral, and aldehydes related to lipid degradation such as (E)-2-pentenal and (E)-2-undecenal, which were not present in BC + W substrates. Preliminary statistical tests with unsplit data (data not shown) yielded significant effects (*p*-value < 0.05) for all variables, overestimating the effect of SSF in BC + G substrates. In BC + W substrates (Fig. 3d–f), both the fungus and SSF time had a significant effect on the odor profiles (*p*-value < 0.001), and a significant interaction effect between these variables was identified (*p*-value = 0.035). Comparing the fermented substrates to those at time 0, SSF increased the intensity of the cereal, malt extract, molasses, and fermented categories. AO and RO SSF achieved the highest increase, while NI didn’t affect the substrate as significantly. The positive effect of SSF over the odor profile had a maximum at 48 h of SSF, as after 72 h the intensity of the cereal, malt extract and molasses categories were significantly lower (*p*-value < 0.05). Comparable trends have been observed in traditional SSF applications. For example, soybean fermentation with RO to produce tempeh is typically limited to 40–50 h, since prolonged incubation results in mycelial senescence, lipid oxidation, and the accumulation of bitter-tasting amino acids that compromise sensory quality^49^. Similarly, the fermentation of peanut press cake, okara, and tapioca waste with NI to produce red oncom is generally restricted to 24–48 h, as longer times cause physical compaction of the substrate and the development of bitter notes^13^.

In BC + G substrates (Fig. 3a, b, and c), neither the fungus nor SSF time had a significant effect on the odor profiles (*p*-value > 0.05), but a significant interaction effect between these variables was identified (*p*-value = 0.034). AO and NI SSF decreased the intensity of the rancid, seaweed, grass, and sweet grass categories over time, with AO achieving the highest impact after 72 h and NI after 48 h. These findings also have practical implications for optimizing AP production. Previous work showed that crude protein content reaches its maximum after 48 h for RO, but only after 72 h for AO and NI^25^. In BC + W, this aligns with the point of maximum positive sensory impact, suggesting that RO SSF can be stopped at 48 h to achieve both an optimal odor profile and increased protein content without loss of yield. For AO and NI, however, it remains to be determined whether extending fermentation to 72 h provides sufficient sensory benefit to justify the longer processing time, or if 48 h represents the most efficient compromise. As each species generates different odor profiles, there is no single “optimal” fungus; rather, each species offers different trade-offs between protein enrichment and odor development, making them suitable for different applications.

The presence of PRG protein in the BC substrate masked the cereal, malt extract, and molasses categories from the BC + G substrates (Fig. 3a–c), underpinning the masking effect of certain VOCs and their impact on human perception^50^. Fermented was the only category present in all samples, primarily associated with the same musty and moldy descriptors in both substrates. Only NI SSF resulted in the crossover of the odor categories between BC + W and BC + G substrates, being capable of surpassing the masking effect of PRG.

### Comparison of predicted and measured odor profiles

**Fig. 4:**
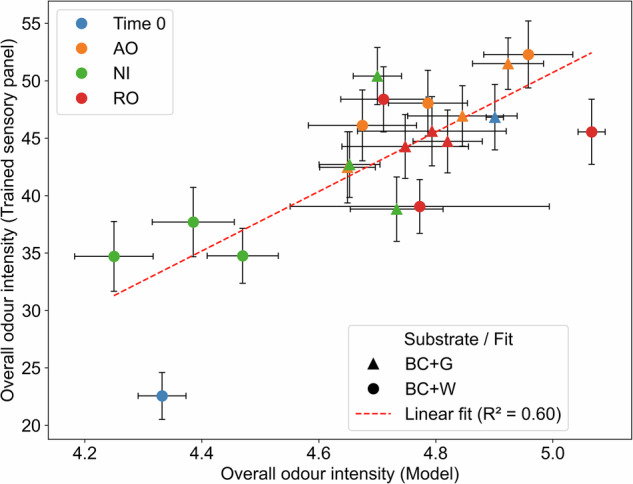
Predicted versus perceived odor intensity. Predicted overall odor intensity versus perceived overall odor intensity by trained sensory panelists for all samples of solid-state fermentation (SSF) with Aspergillus oryzae (AO), Neurospora intermedia (NI) and Rhizopus oligosporus (RO) in bread crusts + water (BC + W) and bread crusts + perennial ryegrass (BC + G) substrates. Points represent the mean value of both variables, and lines represent a standard error (*n* = 3 for the model data and *n* = 13 for the panel data).

Analysis of the odor profiles predicted by the model and scored by the sensory panel reveals statistically supported relationships, alongside the model’s strengths and weaknesses. Figure 4 shows a significant positive correlation (*R* = 0.776, *R*^2^ = 0.603, *p*-value < 0.001) between the logarithmic predicted overall OI of the model and the perceived overall OI of the QDA. The fit illustrates the scale of the model, where a 4.2 to 5.1 range in the logarithmic scale is contrasted with a 25−55 scale by a trained panel. This trend serves as a validation of the Weber-Fechner law regarding the non-linear behavior of human smell, as shown previously in other applications^20^. The values for unfermented BC + W stand out as an outlier in the regression as the lowest OI (panel score of 25). This could have been due to the relatively higher intensity of every other sample, either with PRG and/or fermented, which would affect the intensity perception^47^.

**Fig. 5:**
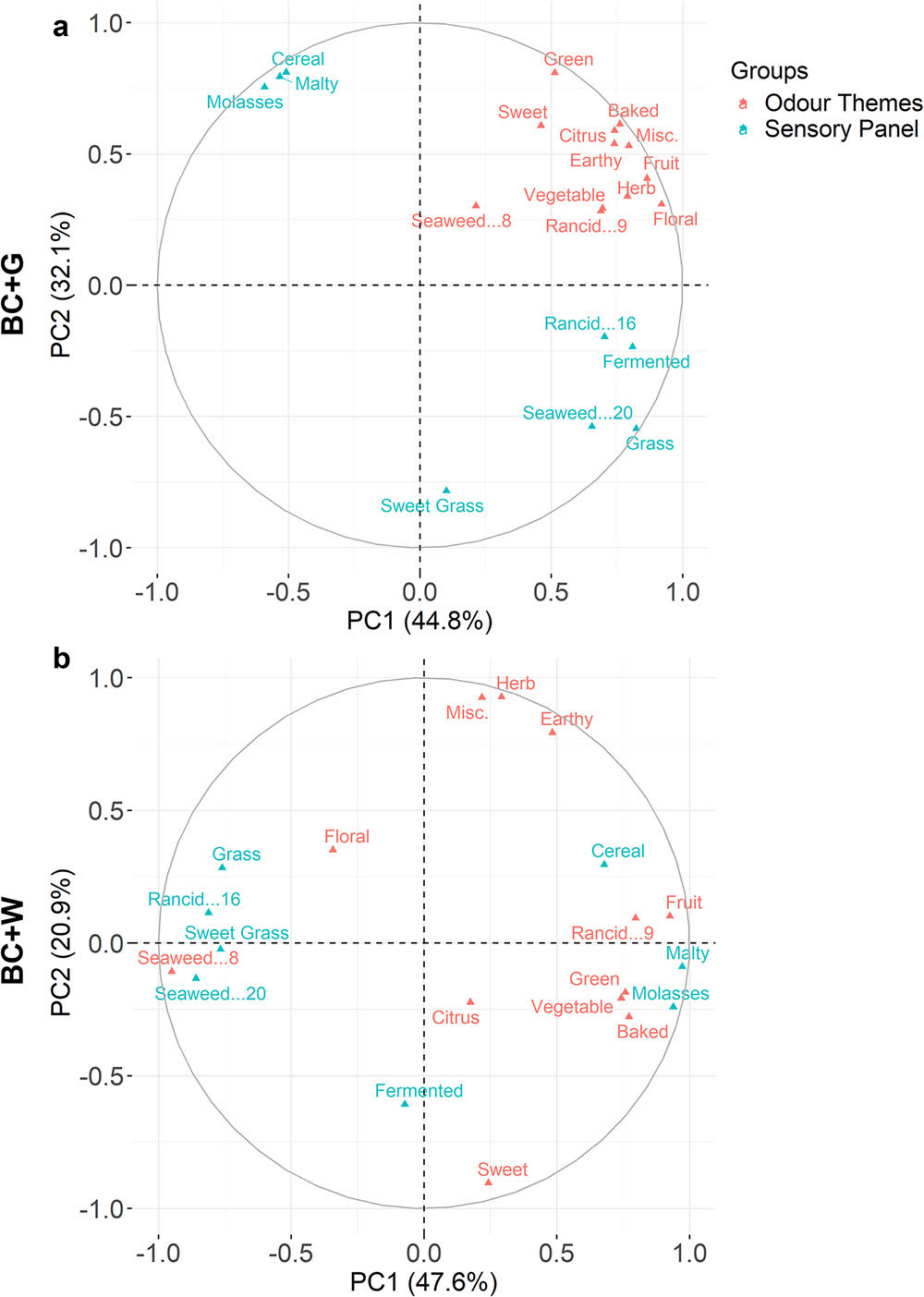
Multiple factor analysis of odor profiles. Variable scores of the multiple factor analysis (MFA) of the predicted odor profiles (odor categories, in red) and the identified odor profiles from trained sensory panel (sensory panel odor categories, in blue) for samples of solid-state fermentation (SSF) with Aspergillus oryzae (AO), Neurospora intermedia (NI) and Rhizopus oligosporus (RO) in **a** bread crusts + perennial ryegrass (BC + G) substrates and **b** bread crusts + water (BC + W).

Figure 5 shows the variable scores of the multifactor analysis (MFA). This data enables the comparison of the predicted and perceived odor profiles by analyzing the proximity of the odor categories between the two datasets. The first two principal components (PC1 and PC2) of the MFA explain 76.9% of the variation in BC + G substrates (Fig. 5a) and 68.5% in BC + W substrates (Fig. 5b).

In the BC + G samples (Fig. 5a), the alignment between predicted and perceived odor profiles is limited. All the predicted odor categories cluster tightly in the upper-right quadrant (PC1 + , PC2 + ). In contrast, the QDA data exhibits a split distribution, with grass and SSF-related categories located in PC1+ and BC-related categories in PC1-. This suggests that PC1 captures the contrast between grassy and baked/sweet odors exclusively from the QDA results, and ultimately that the model incorrectly predicts the intensity of BC-related odors in the presence of PRG.

In the BC + W samples (Fig. 5b), the connection between the predicted and perceived odor profiles is notably improved compared to BC + G. In PC1-, QDA categories such as grass, sweet grass, rancid, and seaweed closely align with the model’s predicted seaweed and floral category, driven mainly by the concentration of dimethyl sulfide and geranyl butyrate in NI SSF, which are the main BC + W samples where these categories were affected. Other unpleasant categories such as green, rancid and vegetable are opposite to this group due to the presence of related descriptors in VOCs common across all samples, regardless of PRG. Particularly, lipid degradation compounds such as nonanal, (Z)-4-heptenal, (E)-2-pentenal, (E,E)-2,6-Nonadienal, 2-hexenal, 3-hexenal and hexanal, are ubiquitous to all samples. In PC1 + , QDA BC-related categories, such as malty, cereal, and molasses, align with the model’s baked and fruit categories, driven mainly by the aldehydes identified as KFOs. PC2 captures the effect of specific fungi over the model’s predictions, which were not identified by the QDA, such as the changes in the sweet category by AO and RO SSF, and the changes in the herb and earthy category by RO SSF.

In terms of the predicted (Fig. 2) and QDA (Fig. 3) profiles, the most notable common trend in BC + G was the reduction in the intensity of the PRG-related odors throughout SSF. Both the model’s predictions (seaweed, green, vegetable, and rancid) and the QDA results (sweet grass, grass, seaweed, and rancid) showed a reduction over time. In BC + W, changes in the predicted baked and sweet categories were also seen in the QDA through increased intensities in cereal, malt extract, and molasses, with AO SSF achieving the highest intensities. Some predicted odor categories that were not explicitly included in the QDA vocabulary may still have influenced the perceived profiles. For example, while NI SSF was not predicted to exhibit stronger sweet notes, it was predicted to have an increased fruit intensity, which the panelists might have perceived within the molasses category, as fruity notes exist within the same sensory dimension^51^. Similarly, the predicted increase in floral intensity could also explain the heightened sweet grass and grass intensities observed in the QDA. In the case of RO SSF, the predicted increase in herb intensity may correspond to the grass notes perceived by the panel. In all SSF, the increase in the QDA category fermented could not be directly compared with any single category in the model; however, it may be related to the concurrent increases in predicted earthy, vegetable, herb, and green categories, which together could synergistically contribute to the overall perception of fermented. Additionally, other compounds with relevant odors in the molasses category might not have been identified within our experiments but could be an important part of the profile, such as the hydroxyfuranones sotolon, furaneol and homofuraneol, which are polar sugar degradation compounds formed from Maillard reactions^52^.

### Limitations and future work

While the modeling exercise enabled comparisons between model predictions and QDA results and allowed the identification of KFOs with potential for further exploration, several limitations need to be addressed. Importantly, modeling should be seen as an iterative process, and this work represents an initial step toward developing a novel mechanistic framework.

The selection of the Weber-Fechner law as the model for this model was based on its simplicity and previous successful approximations to mathematically measure the odor intensity of mixtures based on their chemical composition^18,27^. Other models, such as Stevens’ power law^19^, have been shown to better fit odor intensity data^20^, but due to the lack of available literature on either Weber-Fechner coefficients (${\rm{a}}$) or Steven’s power law index (${\rm{n}}$) for most VOCs identified in this study, it was deemed more appropriate to simplify Weber-Fechner law’s with ${\rm{a}}=1$ as the slope for all VOCs. Future work should address the measurement of these parameters, as it would increase the accuracy of the predicted models. Additionally, odor thresholds should be measured whenever possible within comparable conditions for all VOCs, or at least the most impactful KFOs, as the identified variance in the literature data, and the error added to the model by transforming thresholds measured in other mediums (e.g., air, alcohols and oils) negatively impacts the accuracy of the model^27^.

The model did not incorporate chemical interactions between VOCs, thus not considering potential synergistic and antagonistic effects, which was particularly significant in the presence of PRG, nor perceptual effects in mixtures such as suppression and configural perception. The complexity of these effects has been reported in binary^53^ and tertiary mixtures^54^, and modeled through machine learning models that incorporate hardcoded constraints from expert flavorists^55–57^. Expanding these interactions into real food systems with hundreds of VOCs remains a daunting task, as the mechanistic rules that govern these interactions are poorly understood. Future work should combine machine learning methods with mechanistic modeling to incorporate both psychophysical and fuzzy logic constraints.

The categorization of odor descriptors and categories was based on correlation analysis and expert input, but the use of 1% ethanol solutions on strips to define descriptors imposed limitations. Ideally, descriptors should be derived from authentic compounds assessed at their odor threshold concentrations, in contextually relevant substrates, to avoid biases introduced by concentration effects or matrix effects. Additionally, broad descriptors such as “fermented” risk masking underlying differences in perception, and future panels should be designed to separate these into more specific attributes.

The semi-quantitative nature of SPME GC–MS introduced significant variability in measured VOC concentrations. This could be improved by employing calibration curves for individual compounds (particularly KFOs), labeled standards dosed into a suitable bland matrix and complementary columns (e.g., DB-5 with polar columns). Molecular sensory science techniques, such as GC–O, should also be integrated to strengthen the link between detected VOCs and odor perception.

While the trained sensory panel provided essential validation, variability is inherent to sensory data^58^ and could have been reduced by tailoring training more specifically to broad descriptors (e.g., “fermented”), which were found to be contrasting with the mechanistic model after the panel had been completed, or by separating contrasting substrates (e.g., BC + W vs BC + G). Future studies may benefit from applying the approach to simpler food matrices, such as wine or yogurt, where extensive comparative datasets of VOCs and KFOs exist. This would reduce complexity and improve the precision of the mechanistic model.

Overall, this research demonstrates the potential of a mechanistic psychophysical model to predict trends in the odor intensity and odor profile of foodstuffs. The methodology enables the semi-quantitative identification of KFOs, allowing patterns in the data to be recognized and providing insights into the main contributors to an odor profile. While the model shows statistically supported correlations with trained sensory panels, its predictive accuracy is limited by several factors. These include the omission of synergistic and antagonistic interactions among VOCs, the need for more precise quantification methods, the lack of complete odor threshold data and refined modeling coefficients, and the inherent complexity of the substrates studied. Future work addressing these limitations, particularly by applying the model to simpler food matrices, could substantially improve its robustness. Ultimately, mechanistic models such as this offer an interpretable foundation that can be further developed and potentially integrated with advanced computational approaches to support food formulation and process optimization.

From a biological perspective, the KFOs synthesized during SSF are likely to drive odor profile shifts in the final products. In most cases, the production or consumption of these compounds aligned with observed sensory changes; however, specific fungal effects, such as the elevated fruity intensity of NI or the earthy intensity of RO, could not be fully reconciled with the panel data. These findings highlight the need for further investigation, particularly into substrate-specific effects such as those arising from PRG. The identification of fungi-specific KFOs also provides a rational basis for selecting microbial strains tailored to desired odor outcomes in food applications.

## Methods

### Raw materials

For SSF, phosphoric acid (food grade, 85%), potato dextrose agar (PDA), and Tween 80 were purchased from Merck Life Science UK Ltd. (Dorset, UK). Bread crusts (BC) from surplus wheat flour loaves seeded with malted bread were provided by Bradgate Bakery (a division of Samworth Brothers Ltd, Leicestershire, UK) and manually cut into 1 cm³ cubes.

For solid-phase microextraction (SPME) gas chromatography-mass spectrometry (GC-MS), a divinylbenzene/carboxen/polydimethylsiloxane (DVB/CAR/PDMS) fiber was purchased from Supelco (Nottingham, UK), and a J&W DB-5MS column from Agilent Technologies LDA (Stockport, UK). To calculate authentic linear retention indexes (LRI) in the DB-5MS column, 2,3-dimethylpyrazine (99%), 2,5-dimethylpyrazine (99%), 3-methylfuran (98%), ethylpyrazine (98%), 2-furfural (99%), and methylpyrazine ( > 99%) were purchased from Acros Organics (Thermo Fisher Scientific) (Geel, Belgium), 3-methylbutanal (98%) from Alfa Aesar (Thermo Fisher Scientific) (Haverhill, USA), 2-ethyl-3,6-dimethylpyrazine ( > 95%) from AromaLAB GmbH (Planegg, Germany), 2-pentylfuran (98%) from Avocado Research Chemicals Ltd. (Heysham, UK), 2,6-diethylpyrazine (0.1% in 1,2-propanediol) from Dalgety plc (Genus plc) (Hampshire, UK), (E)-2-decenal (97%), (E)-2-heptenal (98%), α-copaene ( > 90%), and p-xylene ( > 99%) from Fluka (Honeywell) (New Jersey, USA), β-phenethyl acetate (98%), eugenol (99%), and ethyl hexanoate (99%) from Givaudan SA (Vernier, Switzerland), hexanol ( > 98%), 2-heptanone (99%), ethyl decanoate ( > 99%), ethyl octanoate ( > 99%), 2-methylbutyl butanoate ( > 95%), and 2-hydroxy-3-methyl-2-cyclopenten-1-one (98%) from International Flavors & Fragrances Inc. (New York, USA), cubebol (75%), α-santalene ( > 99%), (Z)-α-bisabolene (90%), and β-bisabolene (90%) from Isobionics B.V. (BASF) (Geleen, Netherlands), 1-penten-3-ol (98%), 3-methyl-2-formylthiophene (90%), and 5-methyl-2-formylthiophene (98%) from Lancaster Synthesis Ltd. (Thermo Fisher Scientific) (Haverhill, USA), α-terpinyl acetate (95%), citronellyl butyrate (85%), ethyl lactate (98%), geranyl butyrate ( > 95%), methylisoeugenol ( > 98%), neryl acetate (90%), and santalol ( > 80%) from Mane Ltd. UK (Derby, UK), 2-isopropylpyrazine (99%) from Merck Life Science UK Ltd., 2-ethylfuran (97%), 2-vinylfuran (95%), (E)-2-pentenal (95%), 2-furanmethanol (99%), 2-methylfuran (99%), and 1-pentene-3-one (97%) from Oxford Chemicals Ltd. (Hartlepool, UK), (Z)-nerolidol (98%) from PFW Aroma Chemicals B.V. (Barneveld, Netherlands), 5-methyl-2-phenyl-2-hexenal ( > 96%) from Riverside Organics Ltd. (Northwich, UK), 2-butanone (99%), 3-methyl-2-butenal ( > 97%), 3-methylbutanol (98%), 2,3-butanedione (97%), 2-methylbutanoic acid (98%), 3-methylbutanoic acid (99%), phenylacetaldehyde (10% in ethanol), 2-methylbutanal (98%), dimethyl sulfide (99%), methional (97%), phenylethyl alcohol (99%), 2-hexenyl acetate ( > 98%), (E)-2-nonenal ( > 93%), 2-acetylfuran (99%), 1-furfurylpyrrole ( > 98%), butanol ( > 99%), 2-methylbutanol (98%), 2-formylpyrrole (98%), octanol ( > 99%), 2-methylpropanol (99.5%), β-cyclohomocitral ( > 75%), (E,E)-2,6-nonadienal ( > 93%), (E,Z)-2,6-nonadienal ( > 92%), 2-acetylpyridine ( > 99%), 2-acetylpyrrole (99%), 2-butenal ( > 99%), 2-ethyl-5-methylpyrazine (98%), 2-hexen-1-ol ( > 95%), p-vinylguaiacol ( > 98%), 2-methyl-2-cyclopentenone (98%), (E)-2-octenal (94%), (E)-2-undecenal ( > 90%), (Z)-3-hexen-1-ol ( > 98%), (Z)-3-hexenal (50%), 3-hydroxybutanone ( > 96%), 3-methyl-2-butanone (99%), (Z)-4-heptenal (99%), 2-isobutyl-4-methyl-1,3-dioxolane (97%), 4-vinylphenol (98%), 6-methyl-5-heptene-2-one (99%), 5-methyl-2(3 h)-furanone (98%), 6,10-dimethyl-5,9-undecadien-2-one ( > 97%), 2-methyl-6-heptanone ( > 98%), alpha-humulene ( > 98%), α-phellandrene ( > 85%), α-terpineol (90%), benzyl alcohol ( > 99%), β-cyclocitral ( > 90%), caryophyllene ( > 80%), dimethyl disulfide ( > 99%), ethyl acetate ( > 99%), hexanal (97%), linalool (97%), nonane (99%), p-cymene (99%), pentanal (97%), methylpropanal ( > 99%), 2-methylpropanoic acid (99%), propyl propanoate (99%), safranal ( > 88%), terpinolene ( > 90%), heptanol (98%), 2-methyl-2-butenal ( > 99%), 2-octanol ( > 97%), α-cyclocitral (95%), d-limonene (99%), ethyl 2-butenoate (98%), and (E)-5-methyl-2-isopropyl-2-hexenal ( > 95%) from Sigma-Aldrich (Merck Ltd.) (Missouri, USA), nonanal (96%) from Synergy Flavors Inc. (Illinois, USA), pentanol ( > 99%), 2,3-butanediol 1 ( > 97%), 2,3-butanediol 2 ( > 97%), (E)-2-hexenal ( > 97%), acetic acid ( > 99%), benzaldehyde ( > 98%), and ethyl (E)-2-hexenoate ( > 97%) from Tokyo Chemical Industry Co., Ltd. (Tokyo, Japan), and 2,3-pentanedione (97%) from Thermo Fisher Scientific Ltd. (Paisley, UK). 3-Methylbutyl pyrrole, 4,5-dimethyl-2-isopropyl-1,3-dioxolane, and 4,5-dimethyl-2-isobutyl-1,3-dioxolane were synthesized at Reading University, Reading, UK^59,60^.

### Chemical composition analysis

The moisture content of the samples was determined according to the AOAC method 930.15^61^. Crude protein content of the unfermented substrates was determined using the Dumas combustion method in a Vario MAX Cube CN analyzer (Elementar, Stockport, UK). A conversion factor of 6.25 was used to convert total nitrogen to crude protein content.

### Perennial ryegrass harvesting and pilot-scale processing

Perennial ryegrass (PRG) was seeded in 2022 in a farmed experimental plot at Aberystwyth University in Aberystwyth, UK and harvested in June 2023. Freshly harvested PRG (1.5 metric ton) was screw pressed at pilot scale in a CP-10 screw press (Vincent corporation, Tampa, USA) to obtain 610 L of grass juice (GJ) with a pH of 6.00, and frozen at -20 °C in 3 L aliquots to avoid the growth of undesirable microorganisms. The resulting GJ had a dry matter (DM) content of 5.13% and a crude protein (CP) content of 16.0% DM. GJ (555 L) was clarified using a clarifying decanter SCE 205 (GEA Mechanical Equipment UK Ltd, Milton Keynes, UK) at 5590 rpm, with an internal speed differential of 12 rpm, to obtain 510 L of clarified GJ with a DM content of 0.99%, subsequently adjusted to pH 3.5 with H_3_PO_4_. The clarified GJ was centrifuged in a Scout separator SSE 10 (GEA Mechanical Equipment UK Ltd, Milton Keynes, UK) at 21000 g to obtain 25 kg of wet protein-rich precipitate with a DM content of 21.9%, which was freeze-dried achieving a grass solid (GS) with final DM content of 85.0% and CP content of 40.1% DM.

### Microorganism and inoculum preparation

The fungal strains *Rhizopus microsporus* var. *oligosporus* (tempeh) (RO) and *Aspergillus oryzae* (barley koji) (AO) were purchased from fermentationculture.eu. *Neurospora intermedia* (CBS 131.92) (NI) was purchased from the Westerdijk Fungal Biodiversity Institute (Utrecht, Netherlands).

Fungal spores were preserved in 10% glycerol solution at -80 °C and in PDA plates at 32 °C. After 5 days of growth at 32 °C, spores were harvested from PDA plates by rinsing with a total of 50 mL sterile distilled water (0.03% Tween 80), added in two successive 25 mL portions, and counted in a hemocytometer to 1 × 10^7^ spores/mL and SSFs were inoculated at 2 × 10^5^ spores/g substrate.

### Solid-state fermentation

SSF experiments were conducted in sterile 20x8x5 cm aluminum trays according to Sandoval et al.^24^. Briefly, 100 g of BC, with an initial CP content of 15.9% DM, were either adjusted to a moisture content of 56% (v/w DM) with sterile distilled water (W) to make up the BC + water (BC + W) substrate or supplemented with GS to a CP content of 24.5% and adjusted to a moisture content of 56% with GJ to make up the BC + PRG (BC + G) substrate. Each fungal strain was inoculated into the BC + W and BC + G substrates, and incubated for 24, 48 or 72 h at pH 3.5 (adjusted with H_3_PO_4_) and 32 °C. After the fermentation time, the experiments, and non-fermented samples of the BC + W and BC + G substrates, were dried in a vacuum oven at 45 °C, milled to a fine powder and then stored at 4 °C for further analysis. Samples were dried in these conditions to reduce the loss of VOCs and the generation of artifacts unrelated to fungal metabolism. Each experiment was done in biological triplicate providing a total of *n* = 60 experiments.

The composition of BC, GJ, and GS, and the unfermented mixed substrates BC + W and BC + G can be seen in Table 3. Prior work has established the efficacy of SSF in enhancing the protein content of substrates, with AO achieving the highest increase, followed by RO and then NI^25^. This enrichment is largely attributed to the depletion of carbohydrates, which increases the relative protein content, as well as fungal metabolism which alters the amino acid profile. These results served as a biochemical basis for subsequent analyses of secondary metabolite production.

**Table 3:** Composition of unfermented solid-state fermentation (SSF) substrates. Data is shown as the mean value and a standard error

Material	H (%)	CP (%DM)
Surplus bread crusts	20.8 ± 0.5%	16.8 ± 0.2%
Grass juice	94.9 ± 0.4%	16.0 ± 0.7%
Dry grass solids	15.0 ± 0.5%	40.1 ± 0.5%
Bread crusts + water	56.0%*	16.8%*
Bread crusts + perennial ryegrass	56.0%*	27.0%*

*Values from mass balance of components.

*H* Moisture, *CP* Crude protein, *DM* Dry matter.

### Solid-phase microextraction

The VOCs were extracted by SPME using a CTC-CombiPal auto-sampler (Zwingen, Switzerland) equipped with a DVB/CAR/PDMS fiber. For each experiment, 1.00 ± 0.01 g of dried and milled sample was mixed in an 18 mL glass vial with 3.00 ± 0.02 mL of deionized water (to allow use of an internal standard), 8 ${\rm{\mu }}$L of the internal standard (100-µg/L aqueous solution of 2-isopropylpyrazine) was added, and the vial fitted with a screw cap and a PTFE-lined septum. After equilibration at 50 °C for 20 min, the SPME fiber was exposed to the headspace above the sample for 20 min at 50 °C. Each biological replicate was measured once.

### GC-MS analysis of SPME extracts

After extraction, the SPME device was inserted into the injection port of a 7890 A GC equipped with a J&W DB-5MS column, coupled to a 5975 C inert XL EI/CI MSD triple-axis MS (Agilent Technologies, Stockport, UK). The contents of the SPME fiber were desorbed at 40 °C in a split/splitless injection port in splitless mode. The injector and detector temperatures were maintained at 280 °C and 250 °C, respectively. After desorption, the oven was maintained at 40 °C for 2 min, and then the temperature was raised at 4 °C/min to 250 °C and held for 1 min. Helium was used as the carrier gas, and the flow rate was set at 2.0 mL/min. Mass spectra were recorded in the electron-impact mode at an ionization voltage of 70 eV and source temperature of 200 °C, with a scan range of 29–500 m/z and a scan time of 0.69 s. The data were controlled and stored by the HP G1034C ChemStation data system.

### Volatile compound identification and quantification

VOCs were identified by comparing each mass spectrum with those from authentic compounds analyzed at Reading University, Reading, UK, or with spectra from the NIST/EPA/NIH Mass Spectral database (Version 2.0 g, 2011). To confirm the identification, the linear retention index (LRI) was calculated for each VOC, using the retention times of a homologous series of C6–C20 n-alkanes and by comparing the LRI with those of authentic compounds analyzed under similar conditions. The semi-quantitative quantification of VOCs was calculated from GC peak areas by comparing with the peak area of the 2-isopropylpyrazine standard, using a response factor of 1.

The identified VOCs were categorized by functional chemical groups (as either alcohols, aldehydes, esters, furans, ketones, organic acids, phenols, pyrazines, pyridines, pyrroles, sulfur compounds, terpenes, dioxolanes or others) and by likely source of origin (as either lipid degradation, Maillard reaction, plant derived, SSF or miscellaneous). Their odor descriptors were identified by doing blind smelling trials of 1% v/v solutions of authentic compounds in ethanol applied to odor strips, where authentic standards were available, with expert flavorists (Table 1). Otherwise, the odor descriptors were taken as reported in The Good Scents Company database^62^ (Table A1).

The odor descriptors were grouped into odor categories by performing a Pearson correlation matrix across the odor intensity of all descriptors in all samples, following the procedure described in the mechanistic modeling of odor profiles (but with descriptors instead of categories) grouping those that showed strong positive correlations (Table A2). Descriptors that were correlated in our VOCs but could belong to different groups in sensory practice were further consolidated into categories, guided by the judgment of expert flavorists and the trained sensory panel (e.g., “fishy” and “seaweed” grouped into the seaweed category, “tomato” and “green” grouped into the green category). The resulting categories were defined as baked, earthy, floral, herb, fruit, citrus, rancid, seaweed, green, vegetable, sweet, and miscellaneous, and used for subsequent modeling.

### Mechanistic modeling of odor profiles

A mechanistic model was developed to estimate the odor intensity (OI) of each sample across the different odor categories. Several assumptions were made to define the model’s boundaries properly.

As dry samples were diluted in water before volatile extraction, VOCs were in equilibrium across three phases: from the aqueous solution into the vial headspace, and then into the SPME fiber, before being desorbed into the GC-MS carrier gas. The efficiency of this mass transfer is compound-specific and varies according to volatility, while the observed chromatographic peak area also depends on compound-specific ionization and fragmentation patterns. To account for variability in volatility, the measured concentrations of each compound ${\rm{i}}$ in each sample ${\rm{j}}$ were adjusted using Henry’s law constant (Eq. 3).

3 $${{\rm{C}}}_{{\rm{i}},{\rm{j}}}=\frac{{{\rm{x}}}_{{\rm{I}},{\rm{j}}}}{{{\rm{H}}}_{{\rm{V}},{\rm{i}}}^{{\rm{CC}}}}\,$$

4 $${{\rm{H}}}_{{\rm{V}},{\rm{i}}}^{{\rm{CC}}}=\frac{1}{{{\rm{H}}}_{{\rm{S}},{\rm{i}}}^{{\rm{CP}}}{\rm{RT}}}$$

where ${{\rm{C}}}_{{\rm{i}},{\rm{j}}}$ is the corrected mass concentration in water, ${{\rm{x}}}_{{\rm{i}},{\rm{j}}}$ is the uncorrected mass concentration in the headspace and ${{\rm{H}}}_{{\rm{V}},{\rm{i}}}^{{\rm{CC}}}$ is the dimensionless Henry’s volatility constant (also known as the air-water partitioning coefficient). The volatility constant ${{\rm{H}}}_{{\rm{V}},{\rm{i}}}^{{\rm{CC}}}$ was calculated according to Eq. 4, where ${{\rm{H}}}_{{\rm{S}},{\rm{i}}}^{{\rm{CP}}}$ is Henry’s solubility constant, ${\rm{R}}$ is the universal gas constant and ${\rm{T}}$ is the room temperature (25 °C). The solubility constant ${{\rm{H}}}_{{\rm{S}},{\rm{i}}}^{{\rm{CP}}}$ was obtained from Sander^28^ or estimated by the EPI suite software (V 4.11) (U.S. Environmental Protection Agency). The correction adjusts headspace measurements to semi-quantitative concentrations in the water phase.

The overall OI of each sample ${\rm{j}}$ was estimated based on the odor activity values (OAV) of each compound ${\rm{i}}$ (Eq. 5) using the Weber-Fechner law (Eq. 6).

5 $${\rm{OA}}{{\rm{V}}}_{{\rm{i}},{\rm{j}}}=\frac{{{\rm{C}}}_{{\rm{i}},{\rm{j}}}\,}{{{\rm{C}}}_{0,{\rm{i}},{\rm{water}}}}$$

6 $${{\mathrm{OI}}}_{{\mathrm{j}}}={\mathrm{a}}\,{\log }_{10}\left({\sum }_{{\mathrm{i}}}{{\mathrm{OAV}}}_{{\mathrm{i}},{\mathrm{j}}}\right)+0.5$$

where ${\rm{OA}}{{\rm{V}}}_{{\rm{i}},{\rm{j}}}$ is the odor activity value of compound ${\rm{i}}$ in sample ${\rm{j}}$,${{\rm{OI}}}_{{\rm{j}}}$ is the odor intensity of sample ${\rm{j}}$ and ${{\rm{C}}}_{0,{\rm{i}},{\rm{water}}}$ is the odor threshold of compound ${\rm{i}}$ in water. Compounds lacking either a reported odor descriptor or odor threshold were excluded from the analysis. The ${{\rm{C}}}_{0,{\rm{i}},{\rm{water}}}$ values were compiled from published sources (Table A1). The Weber-Fechner coefficient ${\rm{a}}$ is not available for most of the VOCs identified and was thus assumed to be 1 for all. In cases where the odor threshold values had been collected in air μg/L, they were transformed to thresholds in water (µg/kg) as shown by Tangyu, et al.^21^ (Eq. 7).

7 $${{\rm{C}}}_{0,{\rm{i}},{\rm{water}}}=\frac{{{\rm{C}}}_{0,{\rm{I}},{\rm{air}}}}{{{\rm{H}}}_{{\rm{V}},{\rm{i}}}^{{\rm{CC}}}}\frac{{{\rm{M}}}_{{\rm{i}}}}{{{\rm{\rho }}}_{{\rm{w}}}{\rm{V}}}$$

where ${{\rm{\rho }}}_{{\rm{w}}}$ is the density of water, ${\rm{V}}$ is the molar volume of an ideal gas at room temperature, ${{\rm{C}}}_{0,{\rm{i}},{\rm{air}}}$ is the odor threshold in air and ${{\rm{M}}}_{{\rm{i}}}$ is the molecular weight.

As most compounds were associated with multiple odor categories (up to ${{\rm{N}}}_{{\rm{i}}}$ odor categories) (e.g. methylpropanal is part of both baked and sweet categories ${{\rm{N}}}_{{\rm{i}}}=2$), it was assumed that each compound contributed a $1/{{\rm{N}}}_{{\rm{i}}}$ fraction of its OAV to each assigned category. For each sample ${\rm{j}}$, the OI in category ${\rm{k}}$ was calculated by adding the contributions of all compounds belonging to that category. The set of such compounds was denoted ${{\rm{I}}}_{{\rm{k}}}$ (Eq. 8)

8 $${{\rm{OI}}}_{{\rm{j}},{\rm{k}}}={\log }_{10}\left({\sum }_{{\rm{i}}\in {{\rm{I}}}_{{\rm{k}}}}\frac{{\rm{OA}}{{\rm{V}}}_{{\rm{i}},{\rm{j}}}\,}{{{\rm{N}}}_{{\rm{i}}}}\right)+0.5$$

The final value ${{\rm{OI}}}_{{\rm{j}},{\rm{k}}}$ represents the odor intensity of each odor category in each sample, serving as the model’s prediction of the odor profile. The values were plotted in radar charts to facilitate comparison with traditional sensory results. Values were plotted as the average of the odor scores calculated from all replicates.

### Trained panelist sensory tests

To verify the findings on the mechanistic model, thirteen screened and trained sensory panelists, trained in accordance with ISO 8586:2023^63^, from the MMR sensory research agency (Wokingham, UK), with a minimum of 6 months experience in sensory evaluation of foods and beverages, participated in quantitative descriptive analysis (QDA). The panel was composed of two men and eleven women ranging from 42−62 years old.

In the initial vocabulary training sessions, the panelists were asked to describe the odor profile of SSF samples, followed by a discussion to work towards a consensus vocabulary. In the subsequent sessions, the vocabulary was confirmed by standardizing odor categories against various references (Table 2) to a final consensus of the odor categories: cereal, fermented, rancid, seaweed, grass, sweet grass, molasses, and malt extract. In the following scoring sessions, the panelists were asked to open the vials, sniff the samples, and score the overall odor intensity, and then score each odor category on unstructured line scales (0−100).

Representative samples of the SSF experiments were selected based on the diversity of compounds found via GC-MS and preliminary results of the mechanistic model. Duplicate SSF samples of RO, AO, and NI in BC + W and BC + G substrates at 24, 48, and 72 h of SSF, and the non-fermented BC + W and BC + G substrates were analyzed for a total of *n* = 40 samples. For analysis, 1 g of each dried and milled sample was weight into a 30 mL screw capped amber glass vials. The duplicates of each sample were analyzed on separate days, in a randomized order, in individual sensory booths. The samples were analyzed across 5 days (8 samples per day) and presented monadically coded with three-digit numbers. Between samples, a time delay (1 min) was applied, and the panelists were supplied with filtered water for refreshment if needed. The sensory data was captured using Compusense Cloud (Compusense Inc., Guelph, Canada).

### Statistical analysis

The trained sensory panel’s performance was monitored by applying one-way ANOVA of the sensory score data (overall odor intensity and each category), with the panelists as a fixed variable (data not shown). Multivariate analysis of variance (MANOVA) was used to analyze the effects of SSF over the odor profiles of the trained sensory panel. Two-tailed one-way analysis of variance (ANOVA) followed by Tukey’s HSD post hoc test was used to analyze the overall OI of the model, as calculated with the Weber-Fechner model (Eq. 6), and the odor categories.

To compare the predicted odor profile of the mechanistic model and the profiles given by the trained sensory panel, multiple factor analysis (MFA) was performed using the FactoMineR package in RStudio 12.0. Variables were automatically standardized (mean = 0, variance = 1) to account for the variance in the orders of magnitude the data. Results were primarily interpreted using the first two principal components (PC) of the variable scores, which accounted for ~70% of the total variance of the data.

## Supplementary information

Supplementary information

Supplementary information

## Data Availability

The data supporting the findings reported herein are available on request from the corresponding author.

## Code availability

All data and scripts used for analysis are available on request from the corresponding author.
