# Supplementary material for: Modelling the odor profile of fungal Solid-State Fermentation
Source: NPJ Sci Food. 2026 Jun 19;10:196. doi: 10.1038/s41538-025-00627-0 (PMC13282479; doi:10.1038/s41538-025-00627-0)
Supplement: Supplementary file 2 — Supplementary information [file 41538_2025_627_MOESM2_ESM.docx]

**Legends of the supplementary data of the manuscript:** “Modelling the odor profile of fungal Solid-State Fermentation

Table A1. Identification ions, linear retention index (LRI), functional group category, source of origin category, odor descriptors, odor categories and odor thresholds in air and water of aroma components detected by SPME GC-MS in solid-state fermented (SSF) surplus bread crusts and perennial ryegrass with *Aspergillus oryzae, Neurospora intermedia* and *Rhizopus oligosporus*.

Table A2: Pearson correlation matrix for the odor intensity of odor descriptors

Table A3. Semi-quantitative mass concentration of volatile compounds detected by SPME GC-MS in solid-state fermented (SSF) surplus bread crusts and perennial ryegrass with *Aspergillus oryzae, Neurospora intermedia* and *Rhizopus oligosporus*.

Table A4. Grouping of odor descriptors and odor categories of components detected by SPME GC-MS.

Table A5. MANOVA effects of solid-state fermentation (SSF) conditions over the trained sensory panel odor profiles.
